# *Candida lipolytica* UCP0988 Biosurfactant: Potential as a Bioremediation Agent and in Formulating a Commercial Related Product

**DOI:** 10.3389/fmicb.2017.00767

**Published:** 2017-05-01

**Authors:** Danyelle K. F. Santos, Ana H. M. Resende, Darne G. de Almeida, Rita de Cássia F. Soares da Silva, Raquel D. Rufino, Juliana M. Luna, Ibrahim M. Banat, Leonie A. Sarubbo

**Affiliations:** ^1^Northeast Biotechnology Network, Federal Rural University of PernambucoRecife, Brazil; ^2^Center of Sciences and Technology, Catholic University of PernambucoRecife, Brazil; ^3^Advanced Institute of Technology and InnovationRecife, Brazil; ^4^Faculty of Life and Health Sciences, School of Biomedical Sciences, University of UlsterUlster, UK

**Keywords:** *Candida lipolytica*, animal fat, corn steep liquor, bioremediation, petroleum, heavy metals

## Abstract

The aim of the present study was to investigate the potential application of the biosurfactant from *Candida lipolytica* grown in low-cost substrates, which has previously been produced and characterized under optimized conditions as an adjunct material to enhance the remediation processes of hydrophobic pollutants and heavy metals generated by the oil industry and propose the formulation of a safe and stable remediation agent. In tests carried out with seawater, the crude biosurfactant demonstrated 80% oil spreading efficiency. The dispersion rate was 50% for the biosurfactant at a concentration twice that of the CMC. The biosurfactant removed 70% of motor oil from contaminated cotton cloth in detergency tests. The crude biosurfactant also removed 30–40% of Cu and Pb from standard sand, while the isolated biosurfactant removed ~30% of the heavy metals. The conductivity of solutions containing Cd and Pb was sharply reduced after biosurfactants' addition. A product was prepared through adding 0.2% potassium sorbate as preservative and tested over 120 days. The formulated biosurfactant was analyzed for emulsification and surface tension under different pH values, temperatures, and salt concentrations and tested for toxicity against the fish *Poecilia vivipara*. The results showed that the formulation had no toxicity and did not cause significant changes in the tensoactive capacity of the biomolecule while maintaining activity demonstrating suitability for potential future commercial product formulation.

## Introduction

Surfactants are chemical compounds that preferentially partition at the interface between phases (gas, liquid, and solid) with different degrees of polarity and hydrogen bonding. They are therefore amphipathic molecules with hydrophilic and hydrophobic moieties in which the polar portion is either ionic (cationic or anionic), non-ionic or amphoteric, and the non-polar portion is often a hydrocarbon chain (Santos et al., 2016). These characteristics allow these compounds to reduce surface and interfacial tensions as well as form micro-emulsions in which hydrocarbons are solubilized in water or vice versa solubilizing water in hydrocarbons. Such properties enable a broad spectrum of potential industrial applications involving emulsification, detergency, lubrication, wetting, foaming, dispersions, or solubilization of different phases (Silva et al., 2014).

Most commercially available surfactants are synthesized from petroleum by-products (Silva et al., 2014). However, environmental concerns mostly driven by consumer demands combined with new regulations aimed at managing the environment have led to the pursuit to find alternative natural surfactants to replace existing products. Various compounds with such tensioactive properties are often synthesized by biological systems such as plants (saponins), microorganisms (glycolipids), and animals (bile salts, skin exudates), which are considered natural surface active compounds (Campos et al., 2013).

Compounds of a microbial origin that exhibit surfactant properties are mainly metabolic by-products of bacteria, filamentous fungi, and yeasts capable of lowering surface tension and exhibiting a high emulsifying capacity are the most predominant type of biosurfactants (Marchant and Banat, 2012). The main types of chemical structures of biosurfactants are glycolipids, lipopeptides, lipoproteins, phospholipids, fatty acids, and polymeric in nature. Biosufactants have numerous advantages over surfactants of a synthetic origin in having lower toxicity, stability under wider ranges of temperature and pH, and ability to remain active at high salt concentrations (Banat et al., 2014).

The oil industry remains the major market for biosurfactants utilization, where they can be used in processes involved with the removal and mobilization of oil residues, bioremediation hydrocarbon contaminated environment, and microbial enhanced oil recovery technology (Silva et al., 2014). The bioremediation of soil and water encounters obstacles associated with the biodegradation of petroleum hydrocarbons, as these hydrophobic compounds bond to soil particles and have a low degree of solubility in water, which reduces their bioavailability to microorganisms and consequently limits the transfer of mass for biodegradation (Souza et al., 2014). The key in the process of enhancing the bioavailability of contaminating oils is the mobilization of the hydrophobic pollutant through the aqueous phase. Thus, the use of surfactants develops as an alternative as a mechanism to enhance the solubility of oils through initiating desorption and the consequent mobilization and solubilization of hydrocarbons facilitating transport, access, and assimilation of these compounds by microbial cells (Burghoff, 2012).

Besides organic pollutants, heavy metals are also found in soil and are considered the inorganic pollutants with the greatest potential risk to humans. Metals ions can exist as fixed or soluble minerals in rocks, sand and soil, or as dissolved ions in water or vapors. Metals can also be attached to inorganic or organic molecules or even attached to air particles. Both anthropogenic and natural activities and processes can emit metals into water and air (Sarubbo et al., 2015).

Surfactants can potentially be used, and have been used, to remediate soils contaminated with metals and oils through desorption, solubilization, and dispersion of contaminants in soil, thereby allowing the removal, collection, or reutilization (Aşçi et al., 2008). The necessity to replace synthetic chemical surfactants with natural compounds however, has motivated studies seeking biological alternatives such as surfactin and rhamnolipids, both of which are bacterial biosurfactants (Barros et al., 2007), and sophorolipids derived from yeasts (Coimbra et al., 2009; Menezes et al., 2011; Albuquerque et al., 2012; Rufino et al., 2013). The ionic nature of these agents as well as their low toxicity, biodegradability and excellent surface properties, make them potential candidates for heavy metals removal from contaminated soil, sediment, and waste water (Sarubbo et al., 2015).

Most known biosurfactants are produced on media containing water immiscible substrates such as oil, fats and liquid, or solid hydrocarbons, although many have been obtained on readily available soluble carbon substrates (Pacwa-Plociniczak et al., 2011). The type of raw material and availability of substrate to produce biosurfactants contribute considerably to the cost of production (estimated to be 10–30% of total cost) (Marchant and Banat, 2012). On the other hand, millions of tons of waste materials (residual pollutants) are either deliberately discarded or accidentally leaked into the environment worldwide every year. Treatment and mitigation processes to reduce or eliminate such contaminant represent a high cost to local governments and industries.

Different species of yeast are described as producers of biosurfactants, such as *Yarrowia lipolytica, Rhodotorula*, and species of the genus *Candida*, especially *C. lipolytica, C. antarctica, C. sphaerica*, and *C. bombicola* (Santos et al., 2016). These species are readily found in nature and have significant industrial value, including applications in the environmental, food, and medical fields (Almeida et al., 2016; Santos et al., 2016). Yeasts of the genus *Candida* have also been successfully used in the production of biosurfactants in our laboratories. The biosurfactant from the yeast *C. lipolytica* UCP0988 described here was previously produced in shake flasks and properties of the produced biosurfactant based on its emulsification activity and its stability with different oils and effect of environmental factors on the emulsification activity and stability were reported (Santos et al., 2013). The optimization of cultural conditions for the biosurfactant production using surface response methodology (SRM) and evaluation of its toxicity were also described, showing promising results (Santos et al., 2014, 2017).

Thus, the aim of the present study was to investigate the application of the biosurfactant from *C. lipolytica* UCP0988 in different remediation techniques of organic and inorganic pollutants generated by the oil industry and propose the formulation of a safe, stable remediation agent.

## Materials and methods

### Materials

All chemicals reagent were of analytical grade. The animal fat used was choice white grease from a bovine processing plant located in the city of Recife (Brazil) and was used without any further processing. Corn steep liquor was obtained from Corn Products from Brazil in the city of Cabo de Santo Agostinho (Brazil). According to Akhtar et al. (1997) and Cardinal and Hedrick (1948), corn steep liquor is 21–45% protein, 20–26% lactic acid, 8% ash (containing Ca^2+^, Mg^2+^, K^+^), 3% sugar, and has a low fat content (0.9–1.2%).

Engine lubricating oil (motor oil) was obtained from an automotive maintenance establishment in the city of Recife, Pernambuco, Brazil. Samples of NBR 7214 standard sand (ABNT, 1982) were used in the heavy metal removal experiments. The sand had a particle size on the order of 0.15–0.30 mm, 0.2% water, a specific density of 2.620 g/cm^3^ and organic matter content of 100 ppm. The sea water used in the removal of motor oil was collected from the municipality of Cabo de Santo Agostinho, state of Pernambuco, Brazil. Water samples were collected and stored in 5-l plastic bottles.

### Microorganism

*Candida lipolytica* UCP0988 was obtained from the culture collection of the Universidade Católica de Pernambuco (Brazil). The microorganism was maintained at 5°C on yeast mold agar slants containing (w/v) yeast extract (0.3%), malt extract (0.3%), tryptone (0.5%), D-glucose (1.0%), and agar (5.0%). Transfers were made to fresh agar slants each month to maintain viability.

### Growth conditions

The inoculum of *Candida lipolytica* was prepared by transferring cells grown on a slant to 50 mL of yeast mold broth. The seed culture was incubated for 24 h at 28°C and agitated at 150 rpm. The yeast was cultivated in a submerged culture in 500-ml flasks containing 100 ml production medium with agitation in a New Bruswick C-24 shaker. The production medium contained 5% animal fat and 2.5% corn steep liquor. The medium was sterilized by autoclaving at 121°C for 20 min (all components were sterilized together). The final pH of the medium was 5.3. The inoculum (1% v/v) containing approximately 10^4^ cells/ml was introduced into chilled yeast medium.

### Biosurfactant production and isolation

Biosurfactant production was performed in a 2-l bioreactor (Tec-Bio-Plus, Tecnal Ltda., Brazil) with a working volume of 1.2 l operated in a batch mode, with controlled pH (5.3) and temperature (28°C). The culture medium was inoculated with a 24-h inoculum and fermentation was carried out at 200 rpm in the absence of aeration for 144 h (Santos et al., 2014). The biosurfactant was recovered from the cell-free broth by cold acetone precipitation, as described by Ilori et al. (2005).

### Surface tension

The surface tension of the culture supernatants obtained by centrifuging the cultures at 5,000 g for 20 min was measured using a Sigma 700 digital surface tensiometer (KSV Instruments LTD—Finland) as described by Santos et al. (2013).

### Screening dispersion test

A quick comparative test method using small vials (25 ml) was used for the visual determination of the dispersant effectiveness of the biosurfactant. The motor oil sample (100 μl) was carefully added to the surface of seawater (20 ml) and a vortex with a depth of 1 cm was created by slow magnetic stirring. The dispersant mixture (5.0 μl), i.e., crude biosurfactant (cell-free broth after fermentation) or isolated biosurfactant at half the critical micelle concentration (1/2 × CMC), at the full CMC and twice the CMC (2 × CMC) was added to the center of the vortex. The stirring rate was immediately increased, maintained at a maximum rate of 2,000 rpm for 60 s and then stopped. The level of oil dispersion in the water was visually estimated after a 1-min rest. Classification A was attributed to the resulting brown-black mixture when all the oil was dispersed in the water leaving no slick at the surface, whereas Classification E was used to describe a complete lack of dispersion, i.e., all the oil returned to the surface a few seconds after the end of stirring, leaving the aqueous phase nearly transparent. Classification B to D represented intermediate situations. All screen tests were carried out at room temperature (Brochu et al., 1986/87).

### Swirling bottle test

A 1-l cylindrical open bottle (diameter: 10 cm) with an outlet valve at the bottom to take samples was used in the dispersion experiment. Samples of 200 ml of sea water were added to the bottle and 2 ml of oil was gently added to the surface of the water with a pipette. The crude or the isolated biosurfactant solution was dispensed in the center of the oil slick in the following proportions of biosurfactant-to-oil: 1:1, 1:2, 1:10, and 1:20 (v/v). The isolated biosurfactant was used at half the CMC, the full CMC, and twice the CMC. The bottle was placed on an orbital shaker table at 28°C to induce a swirling motion in the water content of the bottle. The shaking speed was 150 rpm for a period of 10 min, followed by 1–2 min settling time to allow the larger droplets to return to the surface. Samples were taken after 15 min. The first 2 ml of the sample was removed through the stopcock and discarded and 30 ml of the sample was collected. This sample was extracted three times with hexane, as the biosurfactant is insoluble in hexane. The extract was adjusted. Efficacy was calculated by dividing the concentration of dispersed oil in the water (determined by analyzing the hexane extract) by the total concentration of oil, which depended on the total volume of oil added to the flask (Holakoo, 2001; Jain et al., 2012).

### Removal of motor oil from contaminated cotton cloth

The efficiency of the biosurfactant to remove oil with respect to a commercially available detergent (Soap powder, Asa LTDA, Recife, PE, Brazil) was investigated. The detergent and biosurfactant were individually dissolved in water and their efficiency in removing oil from contaminated cotton cloth was checked individually as well as in combination with the biosurfactant at a 1:1 (v/v) ratio. For such, 3 g of lubricant oil was poured on a 25 × 25 cm cotton cloth and allowed to dry at 40°C for 24 h. To test the oil removal capacity, each piece of cloth impregnated with oil was soaked in flasks containing 100 mL of tap water (control), biosurfactant solutions (cell-free broth and isolated biosurfactant at 1/2 the CMC, the full CMC, and twice the CMC), detergent (sodium lauril ether sulfate at the CMC), and a biosurfactant/detergent solution (1:1 v/v) at their CMC. The flasks were kept on a shaker at 30°C and 100 rpm for 60 min. The post-wash water was used to measure the amount of oil removed from the cotton cloth by extracting it with hexane. The extraction process was repeated three times. The hexane was recovered using a rotary evaporator and the residual lubricant oil was measured gravimetrically (Jain et al., 2012).

### Preparation of contaminated sand with heavy metals

The standard sand was artificially contaminated in the laboratory with a metal solution (Cu(NO3)_2_ + Pb(NO_3_)_2_ + Zn(NO_3_)_2_). The salts were separately dissolved in deionized water to achieve a concentration of 1,000 mg/l and then added together to the sand without pH adjustment. The sand was left in contact with the solution for 3 days in a shaker (200 rpm at 25°C) and then centrifuged at 5,000 rpm for 10 min to remove non-adsorbed metals in the solution. The supernatant was discarded and the contaminated sand was dried in an oven at 50°C for 24 h (Juwarkar et al., 2007).

### Treatment of contaminated sand with heavy metals with biosurfactant

A series of washings was performed using the isolated biosurfactant at 1/2 the CMC, at the full CMC, and twice the CMC as well as the crude biosurfactant (cell-free broth). Distilled water was used as the control. A 1% NaOH solution and 0.7% HCl solution as well as combinations of biosurfactant solutions and cell-free broth with 0.7% HCl or 1% NaOH as additives were also tested. 5.0 g of the contaminated sand were transferred to 125-ml Erlenmeyer flasks and 50 ml of the washing solution were added at the different concentrations described above. The samples were incubated on a rotary shaker (200 rpm) for 24 h at 27°C and then were centrifuged at 5,000 g for 10 min. The supernatants were analyzed for metal concentration using an atomic absorption spectrophotometer (Perkin Elmer AAnalyst™ 800).

### Biosurfactant treatment of synthetic wastewater contaminated with heavy metals

The ability to remove heavy metals in water by the biosurfactant was determined in a synthetic fluent containing Pb and Cd. Biosurfactant solutions at 1/2 the CMC, the full CMC, and twice the CMC were then added separately to 500 and 1,000 ppm solutions of lead nitrate and cadmium nitrate. The metal-biosurfactant precipitate was removed and conductivity of the resulting solution was measured. The conductivity meter (TEC-4MP, Tecnal Ltda., Brazil) was calibrated with deionized water before measuring the conductivity of each sample. All tests were performed in triplicate (Das et al., 2009).

### Formulation of biosurfactant

After fermentation, the broth was centrifuged at 5,000 rpm for 20 min for the removal of the cells. Potassium sorbate (0.2%) was added to the cell-free both with the crude biosurfactant. After the treatment of the crude biosurfactant in accordance with the preservation method, the broth was stored at room temperature (28–30°C) for 120 days, with samples withdrawn at 0, 15, 30, 45, 90, and 120 days to determine stability (Freitas et al., 2016).

### Effect of environmental factors on formulated biosurfactant activity

The effects of the addition of different concentrations of NaCl (1, 3, and 5%), different temperatures (40–50°C) for 60 min and different pH values evaluated after adjustment of the broth pH to 5, 7, and 9 with 6.0 M NaOH or HCl on surface tension and emulsification were evaluated at 0, 15, 30, 45, 90, and 120 days.

### Emulsification activity

The emulsification index was measured using the method described by Cooper and Goldenberg (1987), whereby 2 ml of motor oil obtained from a local automotive manufacturer in the city of Recife, Brazil, or vegetal corn oil was added to 2 ml of the cell-free broth in a graduated screwcap test tube and vortexed at high speed for 2 min. Emulsion stability was determined after 24 h and the emulsification index was calculated by dividing the measured height of the emulsion layer by the total height of the mixture and multiplying by 100.

### Toxicity of formulated biosurfactant against *poecilia vivipara*

Acute toxicity tests were performed using the fish *Poecilia vivipara* as an indicator for the determination of the lethal concentration (LC50) of the formulated biosurfactant for 96 h. The specimens were maintained at 26°C in the laboratory and kept in polyethylene aquaria (capacity: 60 l) supplied with fresh tap water. The fish were fed with commercial fish food (Alcon Basic Ltda, Santa Catarina, Brazil). Water temperature, oxygenation and pH in the aquaria were periodically checked throughout the experiment. After a week of acclimation, the fish were exposed to the formulated biosurfactant.

Assays were performed at the Quarantine Laboratory of Sustainable Mariculture of the Fisheries and Aquaculture Department of the Federal Rural University of Pernambuco (UFRPE), Pernambuco, Brazil. Ten specimens were exposed for 96 h without food or water exchange. Experiments were carried out using a static acute experimental methodology. The animals were kept in 2-l fiberglass boxes with 1.5 l of seawater with salinity of 26 and an average temperature of 27°C under constant aeration and a 12 light/12 h dark cycle. Three dilutions of the formulated biosurfactant (cell-free broth plus 0.2% potassium sorbate) in seawater were tested with three replicates each: 1:1, 1:2, and 1:5 (v/v). Controls contained seawater alone. The result of the experiment was based on determination of lethal concentration for 50% of specimens (LC50) and expressed in terms of the mean mortality of three replicates for each dilution tested with the biosurfactant.

### Statistical analysis

All determinations were performed at least three times. Means and standard errors were calculated using the Microsoft Office Excel 2003 (Version 7).

## Results and discussion

### Screening dispersion test

The results of the screening dispersion test demonstrated that both the crude and isolated biosurfactant dispersed a reasonable amount of oil, with a greater concentration of biosurfactant leading to a greater percentage of dispersion (Table 1). However, it is important to consider that the use of the cell-free broth represents a considerable reduction in production cost of the compound, as described in Santos et al. (2016).

**Table 1:** **Motor oil dispersion by biosurfactant from *C. lipolytica* cultivated in distilled water supplemented with 5% animal fat and 2.5% corn steep liquor using beaker-washing method**.

**Biosurfactant solution**	**Classification**
Crude biosurfactant (cell-free broth)	C^1^
Isolated biosurfactant at ½ x CMC	C^1^
Isolated biosurfactant at CMC	B^1^
Isolated biosurfactant at 2 x CMC	A^1^

1 *A, 100% oil dispersion; B, 75% oil dispersion; C, 50% oil dispersion*.

In a previous study by our research group (Santos et al., 2013), we tested motor oil dispersion characteristics of *C. lipolytica* UCP0988 cell-free broth containing biosurfactants and reported high oil spreading efficiency (54% oil displacement). In another study, the crude biosurfactant from *C. sphaerica* grown in low-cost substrates, on the other hand, showed an oil spreading efficiency of 75% in both screening dispersion test and oil displacement efficiency methods (Sobrinho et al., 2013a).

According to Bai et al. (1997), “dispersion is a process by which a hydrocarbon is dispersed in the aqueous phase as tiny emulsions. Emulsions are not generally thermodynamically stable, but may remain stable for significant periods of time due to kinetic restrictions. Dispersion is related to interfacial tension and surfactant concentration and differs from displacement, which is related only to interfacial tension between the aqueous and hydrophobic phases, with no formation of emulsion.”

### Swirling bottle test

One of the oil spill remediation techniques is the application of dispersants to oil slicks. Dispersants used for this purpose are usually composed of a mixture of surfactants and solvents with some additives designed to enhance the dispersion of oils as well as their removal from contaminated surfaces. Dispersants application reduces the effects of the oil spills as it removes the oil from the surface of water reducing the amount of spilled oil. The dispersion of oil into tiny droplets also increase the surface area of exposure which stimulates biodegradation by indigenous microorganisms (NRC, 2005). The effect of factors such as oil viscosity, mixing energy, and temperature on the efficacy of a dispersant need to be evaluated. The solvent normally contained in dispersants acts as a solution for the surfactant components and serves as a surfactant carrier, enabling penetration into an oil slick.

According to Sorial et al. (2004), the “baffled flask test” developed by the Environmental Protection Agency if the USA has been proposed as a replacement protocol for categorizing oil spill dispersants in a “National Contingency Plan Product Schedule.” Therefore, in the present study a similar experiment was performed for the evaluation of the biosurfactant from *C. lipolytica* as an oil spill dispersant measuring the efficacy using motor oil.

To study the effect of the proportion of biosurfactant to motor oil on dispersant efficacy, tests were carried out with different ratios. In this study, the crude and the isolated biosurfactant without the addition of solvents or additives were tested for 15 min after the simulation of an oil spill in a seawater sample (Table 2).

**Table 2:** **Evaluation of biosurfactant from *C. lipolytica* cultivated in distilled water supplemented with 5% animal fat and 2.5% corn steep liquor as oil spill dispersant (data expressed as mean ± standard deviation)**.

**Biosurfactant/oil ratio**	**Dispersion index (%)**			
	**Biosurfactant (1/2 x CMC)**	**Biosurfactant (CMC)**	**Biosurfactant (2 × CMC)**	**Crude biosurfactant**
1:1	5.01 ± 0.4	15.5 ± 0.6	50.0 ± 0.7	41.0 ± 0.2
1:2	2.06 ± 0.6	6.06 ± 0.5	22.0 ± 0.1	20.0 ± 0.5
1:10	2.02 ± 0.8	3.00 ± 0.1	5.70 ± 0.6	3.50 ± 0.4
1:20	1.00 ± 0.7	2.00 ± 0.3	2.70 ± 0.5	2.40 ± 0.7

The biosurfactant concentration is a critical parameter, since a lower concentration of biosurfactant leads to a smaller amount of dispersion. The dispersant/oil ratio is another critical factor influencing dispersant efficacy. The best dispersion index occurred with a biosurfactant/oil ratio of 1:1 (v/v) with a solution of the biosurfactant twice the CMC (50%), while the crude biosurfactant dispersed ~25% of the oil under the same condition. Similar results were observed for the biosurfactant produced by *C. aphaerica* (Sobrinho et al., 2013a) applied to the swirling bottle test. The biosurfactant used below the CMC was inefficient under the tested conditions. It is likely that the increase in agitation speed allowed greater interaction between the biosurfactant and oil, consequently leading to a greater dispersion percentage. In any case, the results of this test demonstrate that the biosurfactant alone has potential for application as a dispersant, but it is likely that additives will increase the efficiency.

### Motor oil removal from contaminated cotton cloth

The use of biosurfactant as a detergent was tested on cotton cloth samples (Table 3). The performance of the biosurfactant was excellent, removing 70% oil at twice the CMC in comparison to oil removal by the commercially available detergent. The biosurfactant at its CMC was also efficient at removing the oil, while the crude biosurfactant was superior to the isolated biosurfactant at half the CMC. On the other hand, the biosurfactant did not exhibit compatibility with the commercial detergent at a ratio of 1:1 (v/v). Commercially available detergents usually contain an anionic surfactant, water softening components, enzymes, and bleaching agents that helps enhancing the washing performance (Mukherjee et al., 2007). Thus, the addition of these substances could increase the efficiency of the biosurfactant from *C. lipolytica*. In a similar experiment, the biosurfactant from *C. sphaerica* also proved to be efficient in detergency tests since the crude surfactant removed 41% of motor oil from contaminated cotton cloth (Sobrinho et al., 2013a).

**Table 3:** **Removal of motor oil from contaminated cotton cloth by biosurfactant from *C. lipolytica* cultivated in distilled water supplemented with 5% animal fat and 2.5% corn steep liquor using beaker washing method (data expressed as mean ± standard deviation)**.

**Washing solutions**	**Removal (%)**
Distilled water (control)	04.10 ± 0.4
Crude biosurfactant (cell-free broth)	36.00 ± 0.5
Isolated biosurfactant at ½ x CMC	30.20 ± 0.7
Isolated biosurfactant at CMC	48.09 ± 0.4
Isolated biosurfactant at 2 x CMC	70.30 ± 0.6
Commercial detergent	28.07 ± 0.3
Isolated biosurfactant at CMC + commercial detergent at CMC (1:1, v/v)	32.45 ± 0.7

### Treatment of heavy metals contaminated sand

Advances in treatment technologies for heavy metals contaminated soils have increased the interest in finding new washing products, such as anionic biosurfactants capable of bonding to metals and do not pose risks to the environment due to their characteristics of lower toxicity and biodegradability (Maity et al., 2013). The mechanisms for heavy metals extraction by biosurfactants include ionic exchange, chelation, dissolution, precipitation, and associations with contra-ions. Metals are believed to be removed through complexes formation with a surfactant at soil surfaces which are mobilized due to the reduction in interfacial tension and the consequent association with surfactants' micelles. Anionic surfactants are negatively charged and therefore have a good affinity toward metal cations while enhancing better removal due to their capacity to reduce interfacial tension (Marchant and Banat, 2012).

It is important for biosurfactants to remain in the aqueous phase and have minimal interactions with the treated soils. However, when large concentrations of biosurfactant are used to ascertain effective heavy metals removal from soil, sorption to soil particles may occur. Hence, its behavior will inevitably depend on the biosurfactants' molecular characteristics, such as charge, hydrophobicity, and soil characteristics (Sarubbo et al., 2015). Thus, the low-cost biosurfactant we produced using *C. lipolytica* was tested with regard to the removal of copper, lead and zinc contained in samples of standard sand. The standard sand with organic matter content of 100 ppm was used to minimize the interaction of the biosurfactant with the soil and maximize metal-biosurfactant interactions.

Solutions of the isolated biosurfactant at different concentrations [1/2 × CMC (0.04%), CMC (0.08%), and 2 × CMC (0.16%)] were tested to evaluate the removal of metals with and without the formation of micelles, which are efficient structures for the mobilization of heavy metals during soil treatment. Metal removal with the cell-free crude biosurfactant was investigated. The likelihood of increasing percentage metal removal was tested using the surfactant with HCl and NaOH as additives. The additives were employed separately while using distilled water as control. The results of the treatment of sand with the biosurfactant solutions are shown in Table 4.

**Table 4:** **Removal of heavy metals contained in contaminated standard sand by washing solutions (data expressed as mean ± standard deviation)**.

**Treatment**	**Removal (%)**		
	**Cu**	**Pb**	**Zn**
Distilled water (control)	17 ± 1.3	11 ± 1.3	15 ± 1.5
1% NaOH solution	11 ± 2.1	16 ± 0.8	15 ± 1.0
0.7% HCl solution	60 ± 1.4	54 ± 1.5	50 ± 1.3
Cell-free broth	40 ± 1.8	30 ± 1.5	7.1 ± 1.5
Cell-free broth + 0.7% HCl	81 ± 1.6	78 ± 1.8	39 ± 1.9
Cell-free broth + 1% NaOH	53 ± 1.4	49 ± 1.5	5.4 ± 1.7
Cell-free broth + 1% NaOH + 0.7% HCl	40 ± 1.2	30 ± 1.4	5.3 ± 1.3
0.04% biosurfactant solution (1/2 x CMC)	30 ± 1.4	35 ± 1.7	7.1 ± 1.1
0.04% biosurfactant solution (1/2 x CMC) + 0.7% HCl	81 ± 2.0	80 ± 1.1	40 ± 1.3
0.04% biosurfactant solution (1/2 x CMC) + 1% NaOH	39 ± 2.0	40 ± 1.3	6.2 ± 1.1
0.04% biosurfactant solution (1/2 x CMC) + 0.7% HCl + 1% NaOH	38 ± 1.7	47 ± 1.4	5.1 ± 1.3
0.08% biosurfactant solution (CMC)	31 ± 1.4	33 ± 1.6	7.6 ± 1.4
0.08% biosurfactant solution (CMC) + 0.7% HCl	81 ± 0.8	82 ± 1.5	30 ± 1.2
0.08% biosurfactant solution (CMC) + 1% NaOH	45 ± 2.1	33 ± 1.4	6.2 ± 1.2
0.08% biosurfactant solution (CMC) + 0.7% HCl + 1% NaOH	49 ± 1.5	31 ± 1.8	5.1 ± 1.1
0.16% biosurfactant solution (2 x CMC)	30 ± 1.5	35 ± 1.5	6.3 ± 1.4
0.16% biosurfactant solution (2 x CMC) + 0.7% HCl	70 ± 1.6	65 ± 1.5	29 ± 1.2
0.16% biosurfactant solution (2 x CMC) + 1% NaOH	45 ± 1.7	40 ± 1.7	5.1 ± 1.8
0.16% biosurfactant solution (2 x CMC) + 0.7% HCl + 1% NaOH	50 ± 1.9	45 ± 2.1	6.5 ± 1.5

The results demonstrate that, under the herein tested conditions, *C. lipolytica* biosurfactant was more efficient in the removal of copper and lead. The control treatments showed 11–17% metals removal from sand while other treatments achieve >80% removal (Table 4). Ochoa-Loza et al. (2007), stated that different surfactants have varying affinities to different metals and are invariably affected by type and concentration of biosurfactant, interaction with additives (acids or alkalines) and soil characteristics.

Heavy metals removal was not proportional to the increase in the concentration of biosurfactant, remaining around 30% for copper and lead as well as 7% for zinc at the concentrations used (1/2 × CMC, CMC, and 2 × CMC). As seen, zinc was not removed efficiently by the biosurfactant solutions. This metal had an affinity for the acid, which removed 30–40% of the metals when combined with the biosurfactant.

Doong et al. (1998) reported heavy metals removal increasing linearly with surfactants increase at concentrations below CMC and remained relatively constant at concentrations above CMC and depended the type of surfactant, the metals involved and type of soil. The high concentration necessary in some experiments is most often related to biosurfactants' sorption or bonding to the components of the soil particles (Wang and Mulligan, 2009). The acid removed 50–60% of the metals adsorbed to the sand when used alone and this removal percentage increased significantly when the acid was combined with the solutions of the isolated biosurfactant and cell-free broth. The base removed ~15% of the metals and generally increased the percentage of copper and lead removal by the biosurfactant, although it had no positive effect on the removal of zinc when combined with the biosurfactant solutions. The combination of the base and acid together when used with the biosurfactant was also not favorable to the removal of the heavy metals. The results suggest that neutralization of the positive effect of the acid occurred when the base was added to the biosurfactant-acid solutions. It should be stressed that treatment with both alkaline and acidic components may reduce soil fertility and change it chemical composition (Sarubbo et al., 2015). Hong et al. (2002) mentioned that Na^+^ may compete with heavy metals binding to the surfactant and forming Na-surfactant complexes. This may also be the case when using biosurfactant, thereby diminishing metal removal when NaOH used compared to the use of acid. However, França et al. (2015) reported higher Zn, Cr, and Cu removal rates when NaOH was added to a biosurfactant solution derived from *B. subtilis*. A possible explanation for this would be the increase in the solubility of the biosurfactant in the presence of NaOH.

The cell-free crude extract removed 30–40% of lead and copper from the sand, indicating that the crude biosurfactant could be used in the treatment of heavy metal contaminated soils. This would be a considerable advantage, since the downstream process to purify biosurfactant obtained through fermentation could account for 60% of the production cost (Dahrazma and Mulligan, 2007).

The possibility of using biosurfactants for the removal of heavy metals has been shown in laboratory studies (Mulligan et al., 2001). A 4% solution of *Torulopsis bombicola* derived sophorolipids removed 3% of the Cu ions from soil samples and did not remove Zn. The addition of 1% of NaOH to the 4% sophorolipid solution, lead to an increase to 36 and 7% Cu and Zn removal, respectively. The highest removal occurred when 0.7% HCl was added to the 4% sophorolipid solution, achieving Cu and Zn removal rates of 37 and 16%, respectively. Rhamnolipid extracts from *Pseudomonas aeruginosa* in comparison removed 35 and 20% Cu and Zn ions, respectively, when used at a concentration of 12%, whereas a concentration of 2% was able to remove 10 and 5% Cu and Zn, respectively. The addition of 1% NaOH to the 2% surfactant solution led to a significant increase in copper removal from 10 to 28%, but a reduction in zinc removal from 5 to 3%. Mulligan et al. (1999) reported an increase in Zn removal when a 2% surfactin solution was used in combination with an alkaline, whereas Cu removal was unaffected by the presence of NaOH. The combination of the base with a 0.5% rhamnolipid solution favored the removal of both metals (65 and 18%) in comparison to the base alone. On the other hand, 100% copper and zinc removal were achieved with 0.7% HCl in a 4% sophorolipid solution. Heavy metals removal from soil using a saponin (0.1–10%) was reported to be proportional to its concentration (Hong et al., 2002). Dahrazma and Mulligan (2007) also reported heavy metal removal from soil increasing linearly with increased rhamnolipid concentration used and that a 5% solution removed 37% of Cu, 7.5% of Zn, and 33.2% of Ni. This is similar to our results which showed metals removal. The addition of a 0.5% rhamnolipid solution to NaOH increased the removal of copper to 28.3% and Ni to 11.5% in comparison to removal rates achieved with the base alone (1% NaOH). Recently, the biosurfactant from *C. sphaerica* showed removal rates of 95, 90, and 79% for Fe, Zn, and Pb, respectively, from soil samples collected from an automotive battery industry. The addition of HCl increased the metal removal rate when used with biosurfactant solutions at 0.1 and 0.25% (Luna et al., 2016).

### Biosurfactants' ability to bind with heavy metals in aqueous solution

The biosurfactant treatment of waste water contaminated with heavy metals was tested using conductivity measures. The conductivity of the biosurfactant solution at half the CMC was 178 μS/cm and increased to 190 and 198 μS/cm at the CMC and twice the CMC, respectively. This increase was due to the anionic nature of the surface active agent. However, conductivity of the solutions containing cadmium (Cd) and lead (Pb) underwent an accentuated reduction upon the addition of the biosurfactant to the metal solutions due to the chelation/precipitation of the positively charged metals, thereby reducing metal ions in solution and consequently reducing its conductivity (Table 5).

**Table 5:** **Conductivity of metal solutions before and after washing with solutions of biosurfactant isolated from *C. lipolytica***.

**Heavy metal**	**Conductivity (μS/cm) of metal solution**	**Conductivity (μS/cm) after treatment with biosurfactant solutions**		
		**1/2 x CMC**	**CMC**	**2 x CMC**
Cd	510.4	15.40	15.28	12.74
Pb	670.4	21.00	21.32	21.83

The results also demonstrate the efficiency of the biosurfactant at the lowest concentration tested (1/2 × CMC), as only little variation in the conductivity of the metal solutions occurred at the higher concentrations (CMC and 2 × CMC). These results indicate that more micelles led to fewer free ions and conductivity was therefore much less than in the solutions with the absence of biosurfactant. The biosurfactant produced by *C. sphaerica* on industrial residues, on the other hand, showed a different behavior since in a similar experiment higher concentration of this biosurfactant removed metals in greater amounts (Luna et al., 2016). These results show that the efficiency of a biosurfactant will depend on its structure and consequently to its capacity to interact with a specific heavy metal.

### Effect of environmental factors on formulated biosurfactant activity

To offer a commercial surfactant agent, the biosurfactant was formulated and its properties (surface tension, which allows the breakdown of an oil spill, and emulsification, which allows a blend in the form of droplets, to facilitate biodegradation by microorganisms) were evaluated over a 120-day period, thereby estimating the shelf life of the proposed product. Potassium sorbate, a widely used a preservative that inhibits the growth of mold, was added to the biosurfactant at the same concentration used in foods.

The formulated biosurfactant with potassium sorbate was analyzed at different pH values and temperatures as well as different salt concentrations (Tables 6–9). The surface tension of the formulated biosurfactant generally exhibited a small, gradual increase throughout the 120 days of storage in the presence of NaCl with variations in pH and temperature. As the change in tension did not surpass 10 units under the conditions tested, one may presume that the formulation with potassium sorbate did not cause significant changes in the tensioactive capacity of the biomolecule, indicating the possibility of using the biosurfactant under specific environmental conditions of pH, temperature and salinity.

**Table 6:** **Surface tension of biosurfactant formulated with potassium sorbate (0.2%) over 120 days with changes in pH and temperature as well as in different concentrations of NaCl (data expressed as mean ± standard deviation)**.

**Time (days)**	**Surface tension (mN/m)**							
	**NaCl (%)**			**pH**			**Temperature (°C)**	
	**1**	**3**	**5**	**5**	**7**	**9**	**40**	**50**
0	26 ± 1.0	27 ± 1.2	27 ± 1.0	27 ± 1.8	28 ± 1.1	28 ± 1.0	26 ± 1.5	27 ± 1.1
15	33 ± 1.1	34 ± 1.0	28 ± 1.2	33 ± 1.3	33 ± 1.4	35 ± 1.3	27 ± 0.9	29 ± 1.3
30	33 ± 1.3	33 ± 1.5	30 ± 1.5	35 ± 1.3	35 ± 1.0	35 ± 1.2	30 ± 1.5	30 ± 0.9
45	33 ± 1.9	33 ± 1.4	33 ± 1.0	35 ± 1.5	35 ± 1.3	37 ± 1.6	32 ± 1.3	29 ± 1.1
90	35 ± 1.0	33 ± 1.0	33 ± 1.3	35 ± 1.4	37 ± 0.9	37 ± 0.5	32 ± 1.1	29 ± 1.2
120	35 ± 1.5	33 ± 1.1	32 ± 1.2	35 ± 1.1	39 ± 1.1	40 ± 1.0	33 ± 1.3	35 ± 1.1

**Table 7:** **Emulsification of motor and corn oil by biosurfactant formulated with potassium sorbate (0.2%) over 120 days with different concentrations of NaCl (data expressed as mean ± standard deviation)**.

**Time (days)**	**Emulsification (%)**					
	**1% NaCl**		**3% NaCl**		**5% NaCl**	
	**Motor oil**	**Corn oil**	**Motor oil**	**Corn oil**	**Motor oil**	**Corn oil**
0	50 ± 3.0	37 ± 2.1	60 ± 1.9	40 ± 3.5	50 ± 2.3	45 ± 2.0
15	60 ± 2.5	38 ± 2.5	80 ± 2.8	44 ± 2.0	76 ± 2.4	46 ± 2.8
30	88 ± 2.0	50 ± 2.4	85 ± 3.0	47 ± 2.1	95 ± 3.2	46 ± 3.0
45	88 ± 2.0	48 ± 2.5	85 ± 2.4	54 ± 2.5	95 ± 3.0	50 ± 2.7
90	88 ± 3.0	48 ± 3.1	85 ± 2.5	54 ± 3.5	95 ± 2.5	50 ± 3.0
120	88 ± 1.5	48 ± 3.0	87 ± 2.0	54 ± 2.5	95 ± 3.0	50 ± 2.5

**Table 8:** **Emulsification of motor and corn oil by biosurfactant formulated with potassium sorbate (0.2%) over 120 days with different pH values (data expressed as mean ± standard deviation)**.

**Time (days)**	**Emulsification (%)**					
	**pH 5**		**pH 7**		**pH 9**	
	**Motor oil**	**Corn oil**	**Motor oil**	**Corn oil**	**Motor oil**	**Corn oil**
0	80 ± 3.1	45 ± 2.0	50 ± 1.5	50 ± 1.1	50 ± 2.7	45 ± 3.0
15	85 ± 2.1	45 ± 1.8	55 ± 2.5	50 ± 2.5	50 ± 2.3	45 ± 2.3
30	88 ± 2.5	45 ± 2.7	100 ± 1.0	55 ± 2.1	95 ± 2.2	45 ± 1.8
45	88 ± 1.8	55 ± 3.1	100 ± 1.0	55 ± 1.8	95 ± 2.3	45 ± 2.5
90	88 ± 2.3	55 ± 2.2	100 ± 0.5	50 ± 2.3	95 ± 2.1	45 ± 3.0
120	88 ± 1.6	55 ± 1.1	100 ± 1.0	50 ± 3.5	95 ± 1.2	45 ± 3.1

**Table 9:** **Emulsification of motor and corn oil by biosurfactant formulated with potassium sorbate (0.2%) over 120 days with different temperatures (data expressed as mean ± standard deviation)**.

**Time (days)**	**Emulsification (%)**			
	**40°C**		**50°C**	
	**Motor oil**	**Corn oil**	**Motor oil**	**Corn oil**
0	50 ± 2.0	40 ± 1.7	50 ± 2.7	45 ± 3.0
15	50 ± 2.8	40 ± 1.9	60 ± 2.9	45 ± 2.8
30	55 ± 3.0	40 ± 2.4	95 ± 2.7	45 ± 2.7
45	60 ± 2.7	50 ± 2.0	95 ± 3.2	55 ± 2.0
90	60 ± 2.3	50 ± 2.8	95 ± 3.0	55 ± 1.9
120	60 ± 1.7	50 ± 3.0	95 ± 1.7	55 ± 3.0

The emulsification index values in the presence of NaCl, showed some improvement with increase storage time especially after 30 days. Higher salt concentrations had no negative effect on the action of the biosurfactant showing ability to use in saline environments.

The emulsification of corn oil remained practically stable with the change in pH values throughout the storage time, whereas the emulsification of motor oil showed slight increase after 30 days, demonstrating that the interaction between the biosurfactant and oil may be strengthened over time, indicating greater stability of the inter-molecular bonds.

The findings demonstrated that it was possible to formulate a product that remains free of contamination and maintains stability and can be commercialized as an efficient, low-cost, biodegradable agent for use by different industries. The results regarding the formulation of the biosurfactant are difficult to discuss, as the literature on this topic is scarce. Another biosurfactant from *C. bombicola* produced and formulated in our laboratories showed similar results regarding stability (Freitas et al., 2016). Some studies describe the use of spray drying for the conservation of biosurfactants and later application (Saeki et al., 2009). Spray drying has also been effective in the recovery and concentration of a biosurfactants while maintaining their surface activity and the dry product maintained its characteristics and activity during storage at room temperature throughout a 120 day evaluation period (Barcelos et al., 2014).

### Toxicity of formulated biosurfactant

The fish *P. vivipara* belongs to the family *Poecilidae*, which occurs from the United States to Argentina. This species has been used as a bioindicator in the monitoring of aquatic environments due to its sensitivity and response capacity to environmental pollutants (Breseghelo et al., 2004). Acute toxicity tests were conducted with this fish to determine the mean lethal concentration (LC_50_) of the formulated biosurfactant over a 96-h period. The biosurfactant formulated from *C. lipolytca* was considered to have low toxicity, as the *P. vivipara* survival rate was respectively 70, 75, and 95% for biosurfactant/seawater dilutions of 1:1, 1:2, and 1:5 (v/v). Toxicity tests with other biosurfactants produced from *Candida* species have also indicated the absence of toxicity of these biomolecules against marine bioindicators (Sobrinho et al., 2013b; Rufino et al., 2014; Freitas et al., 2016; Luna et al., 2016).

## Conclusion

The present findings demonstrate that industrial waste products can be successfully used in the production of surfactant agents with broad applications in the environmental remediation of organic and inorganic pollutants. Biosurfactant from *C. lipolytica* presented satisfactory results regarding the treatment of sites contaminated with petroleum products and heavy metals. The possibility of commercializing an agent with long-term stability was also demonstrated, making the production process, and application of biosurfactants more viable in the current market of chemical surfactants derived from petroleum.

## Author contributions

All authors contributed in this work. LS conceived and designed the experiments; DS, AR, Dd and RS performed the experiments; IB, RR, and JL analyzed the data and contributed to analysis tools; LS and IB wrote the paper.

## Funding

Funding for this study was provided by the State of Pernambuco Foundation for the Assistance to Science and Technology (FACEPE), the Research and Development Program of the Brazilian National Electrical Energy Agency (ANEEL), the National Council for Scientific and Technological Development (CNPq), and the Federal Agency for the Support and Evaluation of Graduate Education (CAPES).

### Conflict of interest statement

The authors declare that the research was conducted in the absence of any commercial or financial relationships that could be construed as a potential conflict of interest.
